# When more is not merrier: Using wild population dynamics to understand the effect of density on *ex situ* seahorse mating behaviors

**DOI:** 10.1371/journal.pone.0218069

**Published:** 2019-07-02

**Authors:** Heather D. Masonjones, Emily Rose

**Affiliations:** 1 Biology Department, University of Tampa, Tampa, FL, United States of America; 2 Department of Biology, Tufts University, Boston, MA, United States of America; Institute of Animal Science, CZECH REPUBLIC

## Abstract

Seahorses are considered one of the most iconic examples of a monogamous species in the animal kingdom. This study investigates the relationship between stocking density and mating and competitive behavior from the context of the field biology of the dwarf seahorse, *Hippocampus zosterae* (Jordan & Gilbert). Animals were housed in 38 liter tanks at a range of densities and sex ratios (from 2–8 animals per tank), and their reproductive and other social behaviors were monitored from tank introduction through copulation. At low tank densities and even sex ratios but comparatively high field densities, frequency of both mating and competitive behaviors was low in trials. A higher level of males in tanks across all densities increased competition, activity levels, and aggression leading to egg transfer errors and brood expulsion, resulting in lower reproductive success. Across seahorse species, mean and maximum wild densities were consistently lower than those used in *ex situ* breeding, with adult sex ratios that were significantly female biased. However, significant variation exists in wild seahorse densities across species, with higher densities detected in focal/mark recapture studies and on artificial habitat structures than reported with belt transect sampling techniques. Interchange of knowledge gained in both aquarium and wild contexts will allow us to better understand the biology of this genus, and improve reproduction in captivity. Interpreting *ex situ* reproductive behaviors of seahorses within various densities reported from natural populations will help us predict the impact of conservation efforts and increase the likelihood of long-term persistence of populations for this threatened genus.

## Introduction

Maladaptive behaviors have been observed in captivity in animals brought in from the wild, often associated with holding and/or capture stress [1–3]. However, much of the research on behavior in captivity has focused on animals bred in aquaria, with the expectation that either genetic or epigenetic factors were involved in the development of behaviors that lead to lower survival or lower reproductive value in captivity [4,5]. In addition, research exists to suggest that there are behaviors observed in the wild and when placed in that context are adaptive, but become maladaptive in the captive context, especially given the constraints of environments like aquaria [6]. Partnering with researchers studying both the population biology and behavior of animals in the wild is critical to identifying the root of these behaviors and how to manage them *ex situ*.

In syngnathid fishes, field densities of animals have been generally described as low yet variable, especially for seahorses [7,8]. This low population density may affect many aspects of their behavior, especially social behaviors like mating and competition. In addition, it is probably one of the key factors associated with their described monogamous mating system in the wild, observed in both behavioral observations (*H*. *whitei*, [9]) and using genetic tools [10–13], although genetic data indicates that mate switching is more common than originally assumed, at least for *H*. *subelongatus* [11] and *H*. *guttulatus* [13]. Locally high field densities of *H*. *abdominalis* were associated with socially promiscuous behavior, with multiple females in a female-biased population interacting with a single male, but genetic testing confirmed that within a brood, males mate monogamously [14]. More recently, genetic data from wild *H*. *guttulatus* populations varying in sex ratio and seahorse density have shown that within a breeding season, animals are socially polygamous but genetically monogamous, with the potential for mate switching between seasons [13,15]. In both studies, wild-caught individuals maintained in high-density breeding colonies in captivity displayed the potential for more frequent mate switching between broods within a mating season. Thus, density appears to be an important factor influencing the expression of social behaviors in wild seahorses, and potentially an important consideration for the expression of common social behaviors in captivity.

Surveys across the literature indicate that collecting relevant density data from wild populations of seahorses is challenging and varies dramatically by sampling method, as was first identified in Foster and Vincent [8]. Transect sampling is a standard tool used to measure population density in seahorses, but because of their patchy distribution, will underestimate the local density of animals [16]. Field density measurements that include “zeros” (sites with no seahorses) are important to understand their general distribution across the seascape, but because there are many zeros searching for these animals, their local abundance may be much higher (the number of seahorses observed when they are present at a site) [17]. It is this local density that is important from a behavioral perspective. In a study of *H*. *capensis*, comparison of the use of focal study grids (0.22 animals m^-2^) and randomized belt transects (0.0089 animals m^-2^) indicate a dramatic underestimate of the local density of animals with belt transects alone [18]. In addition, density in many populations differs dramatically on a seasonal basis, so timing of surveys is also critical to our understanding of local breeding densities [19,20].

The focus of the present work is to investigate the effects of aquarium holding density on the suite and frequency of courtship and competitive behaviors observed in the dwarf seahorse, *Hippocampus zosterae*. In addition, this *ex situ* behavioral profile will be compared to previous findings observed in the wild for this species and others, in terms of both the animal field density and behaviors. To place this work in context, we conduct a survey of population biology for all seahorse species currently published in the literature to identify the variation in natural population densities and sex ratios and the collection techniques utilized for each study. Finally, recommendations regarding aquarium husbandry, field collection techniques, and implications for conservation to better understand the behavioral context of this genus will be investigated.

## Materials and methods

### Ethics statement

*Hippocampus zosterae* used in these studies were collected periodically from early February through September near either Key Largo, FL (USA; 1994–1996) or in Tampa Bay, FL (USA; 2006–2012). Collection in the Florida Keys was conducted by Aqualand Pets (Miami, Florida) under their Florida Special Activities License (SAL). Animals collected elsewhere in Florida were collected by the authors under SAL # 05SR-902. Both sites from which animals were collected had similar densities of seahorses [21], and recent genetic work indicated interbreeding between Tampa Bay and Florida Keys populations [22]. At the time the research was conducted, neither institution required ethical review of protocols for fish; however, animals were held according to the recommendations in the Guide for the Care and Use of Laboratory Animals of the National Institutes of Health. Following this research, all animal use protocols were approved under University of Tampa AUP #2018–1. No animals were harmed in the study in terms of physical wounds, bruising, or fin damage. Because of the tubular snout with no teeth, seahorses are incapable of inflicting the biting damage associated with other fish species. They do use their tails to hold on to each other, but the authors have never observed death or damage as a result of tail holding in this species.

### Study organism

*Hippocampus zosterae* occurs in shallow seagrass beds from the Gulf of Mexico east through the Bahamas, Cuba and Bermuda [21]. Adult size ranges from 16–55 mm (measured as the linear distance from the top of the coronet to the end of the tail [19,23]). In the lab, females transfer one entire clutch of eggs to a single male, rather than in pipefish, where eggs are produced continuously [24,25]. Egg clutches were counted either from males after eggs were transferred to pouches or from eggs dropped on tank bottom by females, so brood size is likely an underestimate ($\bar{X}$ (SE): 12.5 (1.32) eggs, n = 14; [26]). Five to 30 (15.9 (1.98); n = 29) fully independent young are born after approximately 12 days gestation within the male brood pouch in the lab at 26°C, and pairs usually remate within 4–20 hours of the male’s releasing young [26]. Research from field populations suggests that for a given brood, only one mother contributes eggs, indicating genetic monogamy, but it is still unknown whether pairs mate repeatedly in a long-term monogamous fashion in *H*. *zosterae* [12].

### Experimental conditions and holding facilities

In an attempt to standardize reproductive status, fish were maintained in small (5–8 fish) same-sexed groups (in sexual isolation) for ten days to eight weeks before use in experiments (holding time: $\bar{X}$ = 16 days). This length of time allows for the young from a previous mating to be born. In addition, given seahorses socially monogamous mating system observed in *H*. *whitei* in the wild [9], this time appears to break the pair bond and increase the motivation in both males and females to remate, previously observed in *H*. *zosterae* [26]. All fish were kept in 38 l aquaria prior to and during experiments. Tanks were maintained at an average temperature of 25°C (range 24–27°C) on a 13 hours light/11 hours dark photoperiod, with lighting provided by standard, double bulb, broad spectrum, 122cm shop lights suspended roughly 30cm above tanks along the short axis of the tank. Salinity ranged from 26–33‰ (with seawater made from Instant Ocean [Aquarium Systems, Inc] or Coralife [Central Garden and Pet] salt mix), and artificial seagrass plants were supplied for attachment sites. Fish were fed recently hatched *Artemia* each day immediately after observation (San Francisco Bay brand or Brine Shrimp Direct brand eggs), and supplemented every other day with Selcon (American Marine), a food additive containing highly unsaturated fatty acids.

Data for this study were collected in two locations. The early work (80% of pair trials and 80% of trials with 3 fish; August 1994-August 1996) was conducted at Tufts University (Medford, MA, USA). Animals were housed in the same location in which the study was conducted, inside a fish holding room on the campus (rough dimensions were 3.5 X 4M). The second phase of the work (20% of pair and 3 fish trials, and 100% of the larger group trials; Spring seasons 2006, 2010, 2011, 2012) was conducted at the University of Tampa (Tampa, FL, USA). In both experimental contexts, tanks were isolated from each other, with filtration provided via sponge filters seeded with bacterial starter, which were siphoned and topped off weekly with artificial salt water (from Instant Ocean Salt/Coralife Salt). A statistical comparison of our 1:1 sex ratio treatments between the two locations indicated a difference in variances of activity levels between the two groups (Levene’s test, F_1,33_ = 7.469, p = 0.01), but a nonparametric comparison indicated no difference existed in the activity level between groups (Wilcoxon test, χ^2^ = 2.62, 1 df, p = 0.11). As a result, given the consistency in tank/room setup between the two sites and the consistent responses, we combined data for analysis.

Variations in density and sex ratio used in this study were well below the reported/recommended density conditions for fish [27]. The maximum densities used in this experiment were 10 animals per tank in general housing (1 fish per 3.8L) and 8 animals per tank under experimental conditions (1 fish per 4.75L). Peer-reviewed sources for housing *H*. *zosterae* are scarce, but of the published literature for seahorse species in general, stocking densities down to 0.5 animals per liter have been shown to have no effect on growth and survival [13,28–31]. The lack of specific guidelines for stocking densities for fish is part of the reason this study is important, because there are few studies of this type (Table 1). The study also relates stocking density to data from the wild, to help use that information to understand the potential behavioral and psychosocial effects of density on the successful reproduction of this species and other species.

**Table 1:** Aquarium studies of seahorse species for aquaculture purposes.

Species	Max Field Density	Stocking Density (#/m2)	Tank Sizes	Sex Ratio	Behavior/Study Focus	Ref
***H*. *abdominalis***	0.2	50, 100, 250/140	9 l, 0.02m^2^	8 animals/tank; 1:1, and sex segregated—90 day study	Wrestling rates increase with density; MM, FF courtship observed, no difference in growth	[31]
***H*. *capensis***	0.25	17.4	50 l, 0.12 m^2^	2 animals/tank; 1:1	Photoperiod and temperature effects on breeding	[32]
***H*. *guttulatus***	1.52	20	90 l, 0.29m^2^	6 animals/1:1	Effect of various diets on growth of adult seahorses	[33]
***H*. *guttulatus***	1.52	12/24	180 l, 0.20m^2^	1:1. 3:1, 1:3 ratios explored across range of densities	Lower activity levels seen in low density tanks; competition increased with male density	[34]
***H*. *kuda***	0.73	14	560 l; 0.865m2	12 animals /tank; 1:1; pairs segregated with mesh	Age of parents effect on brood quality	[35]
***H*. *trimaculatus***	NR	2 per l/6-8	2000 l; 4.5m2	14 animals/1:1; males moved to individual tank after copulation	Brood stock nutrition	[36]

**Aquarium studies of seahorse species for aquaculture purposes**, providing maximum animal density (for reference), estimated stocking density (animals m^-2^), tank sizes, stocking sex ratio, and summary of study focus. Studies that focused on juvenile culture and not adult reproduction were excluded. NR–Not Reported.

### Experimental procedure

Daily observations of low density (1 Male : 1 Female; data from both [26] and observations conducted in 2006 and 2010), female-biased (1M:2F), male-biased (2M:1F; latter two treatments from unpublished work described in [26] and observations in 2006) and larger group (2M:2F, 3M:3F; or 3M:4F; observations conducted in 2010, 2011, 2012) experimental treatments were conducted to determine patterns of courtship, mating, and other social interactions in *H*. *zosterae*. On the day before each trial began, wet masses for all fish were measured by first blotting fish dry and weighing them to the nearest 0.01 mg in seawater in a 2 ml plastic vial. Experimental fish used across all treatments ranged from 75–387 mg wet mass, with no more than 50 mg difference in body mass between the smallest and the largest fish used in a trial. Each fish was used in only one trial.

Fish were placed singly into aquaria in random order. Behavioral observations for each trial started immediately after all fish were placed into tanks and continuing until copulation occurred. If copulation did not occur within 7 days, then observations were terminated. Previous observations suggest that if copulation does not occur within a week, it is very unlikely to occur at all [37]. Observations were made continuously during the first 1 to 3 hours after tank lights came on (defined as dawn) on each day before copulation, and from dawn through copulation on the final day. Additional observations were conducted post copulation in some trials, which occurred the first 1–3 hours after dawn. Previous observations have indicated that most seahorse courtship occurs in the morning, except on the day of copulation [37]. Competitive and courtship behaviors were recorded continuously for all fish, in a subset of trials for all treatments. Behavioral observations were recorded using a behavior recorder program written in Visual Basic 6.0 (Microsoft) specifically for recording seahorse behavior patterns for 1:2 and 2:1 treatments, with more traditional observation and recording methods used for pair treatment and larger group treatment.

#### Competitive behaviors

Four competitive behaviors were continuously recorded during observations (behavioral descriptions modified from [26,38]). Snapping (S; S1 Fig) was an instantaneous behavior during which the initiating fish oriented its snout towards the operculum of the receiving fish, rapidly elevating and extending the head in the direction of the receiver. This behavior is associated with a snapping noise (audible with a hydrophone; [39]). The receiving fish often responded to this behavior by darkening its coloration and flattening its body parallel to the bottom of the tank. During holding (H; S2 Fig), the initiating seahorse used its tail to hold onto a portion of the body of the receiving seahorse, including the head. Two fish were considered to wrestle (W; S3 Fig) [26,38], if the held fish began to struggle and the holding fish did not let go for an extended period of time. Intruding (I; S4 Fig) occurred when the initiating seahorse placed its body between a male and female courting pair. Snapping, holding and wrestling could occur between same-sexed or opposite-sexed pairs, but intruding was scored only when observed between courting male-female pairs. Although recorded, because intruding is a behavior that can only be observed in treatments that have more than two fish, it was not included in competitive behavior totals for the purposes of analysis because it would artificially increase rates of overall social behavior in all treatments expect for pair treatments. Many of these competitive behaviors are included in the supplemental video (S1 Video).

#### Courtship behaviors

Four discrete phases of courtship in paired dwarf seahorses have been observed [37], including a number of unique seahorse courtship behaviors (first described by [38,40]). All courtship in dwarf seahorses is accompanied by a rapid and sustained change in coloration (brightening, B) from normal body color (varying from black to white) to a lighter body color over most of the body, excluding portions of the head and dorsal midline (which remain dark). Brightening is an indication of social activity in the tank, and thus activity measurements described in the results are associated with the amount of total observation time that at least one fish spent in brightened coloration.

Phase 1 courtship occurs during the 1 to 2 days preceding the day of copulation, characterized by reciprocal quivering (Q; S5 Fig), during which fish assume an erect body posture, with pectoral fins extended, and rapidly vibrate their bodies from side to side. Pumping (P) is a behavior shown exclusively by male seahorses, and consists of a male opening his brood pouch and repeatedly flexing his tail in a motion similar to that displayed during the expulsion of young. Pumping is observed infrequently on the days before copulation in pairs [26], but is demonstrated by males as early as Phase 2 and used at an indicator of a male’s readiness to mate on the day of copulation in response to female pointing. Courtship phases 2–4 occur on the day of copulation. During phase 2, females first display pointing (Po) a behavior used as an indicator of a female’s readiness to mate and the beginning of the day of copulation. During this behavior, the head is raised upward toward the water surface to form an oblique angle with the main body axis and then lowered to a horizontal position. Males generally respond to female pointing by quivering. Phase 2 is usually followed by a latency period of 23–220 min (median—111 min), during which it is hypothesized that females undergo the last stages of egg maturation and ovulation [37]. In phase 3, males begin to display pointing in response to female pointing. Phase 4 courtship is characterized by the male and female rising (R) repeatedly. During this phase, fish release their respective holdfasts and rise up into the water column facing one another. In a copulatory rise, the female’s genital papilla is placed inside the male’s brood pouch opening, followed by egg transfer, filling the male’s pouch with eggs, into which he releases sperm (S6 Fig). Although the above listed courtship behaviors are described between males and females, the behaviors were also recorded if they occurred between same-sexed pairs or if all multiple fish were engaged in these behaviors simultaneously. Supplemental video demonstrates all of the above behaviors across 4 males and 4 females (S1 Video).

Courtship interactions occurred when at least two fish were within 4 cm of one another (interacting proximity) and both fish exhibited brightened coloration. During courtship interactions, both sexes exhibited a characteristic posture consisting of an erect body with head inclined downward and were generally positioned side-by-side facing the same direction. Courtship occurred in discrete bouts, which began with the first display of any courtship behavior (quivering, pointing, or rising) by any one of the fish and ended when an individual stopped courting and moved out of the side-by-side position.

### Statistical analysis

For the purposes of calculating overall social behavior activity level, time spent “active” was defined as the period of time any animal in the tank was in the brightened coloration state, and/or when any animals were displaying competitive or courtship behaviors, divided by the total observation time that day. Mean number of fish interacting (displaying brightened coloration, competition, and/or courtship) was calculated for every minute of the active period (equivalent to a weighted average of the number of fish active weighted by the fraction of time they spent at that number of interacting individuals). Because fish can engage in brightened coloration individually, active time in tanks can include single fish in the calculation of the average number of fish in interactions. Social behavior activity levels and mean number of fish interacting were compared between treatments using a Kruskal-Wallis test when variances were unequal and ANOVA when assumptions of parametric tests were met. Multiple comparisons were provided by Dunnett’s test using the 1:1 treatment as a control for parametric data and Wilcoxon multiple comparisons for nonparametric data. The overall frequency of aggressive behaviors was calculated for a subset of trials within each group (1:1 –n = 15, 1:2 –n = 14, 2:1 –n = 15, larger groups–n = 12), were investigated relative to the density of males in trials, and were analyzed using Kruskal-Wallis tests because of unequal variances. In addition, the presence/absence of specific behaviors in trials was also recorded for trials in early versus later courtship, and the association between treatment and the presence and absence of these behaviors was investigated using Contingency Table Analysis according to the procedures in [41]. Courtship behaviors between specific individuals were recorded for all trials, and as a result, the number of courtship partners was measured in trials above 1:1 both early and later in courtship. The number of courtship partners both on the first day of courtship and the day of copulation were compared between treatments using Kruskal-Wallis procedures because of unequal variances. To investigate the association between treatment and the various mating success variables (successful transfer of any eggs from male to female, frequency of partial egg transfer, and brood success), contingency table analysis was employed. Statistical analyses were performed using JMP Release 11.2 (SAS Institute Inc).

#### Population density and sex ratio meta-analysis

From a survey of relevant field studies published on seahorse population biology (n = 39 studies), mean and maximum population densities and sex ratio data were extracted for a total of 14 species. Population densities were calculated separately for natural and artificial habitats, and for belt transects (both random and haphazard designs) and focal site studies (including those that use mark recapture surveys to estimate population sizes), when possible from data provided in research studies. Studies were included if they reported requested data directly, provided raw animal numbers and area sampled, or had figures from which data could be mined, and excluded if there was no way to convert either group numbers or density measurement variables to animals m^-2^. Two numeric values provided in S1 Table either indicate that the study had two different methods presented (sampling method), investigated natural and artificial habitats, or data are provided for distant locations. Data from studies matched for species were compared for natural (n = 20 studies and 5 species) and artificial habitats (n = 10 studies) and between belt transect studies (random or haphazard; n = 19 studies and 7 species) and focal study grid/mark recapture studies (n = 11) using Wilcoxon tests, because distributions were neither normal nor were variances equal. The adult sex ratio (ASR) was calculated as the ratio of males:total adults observed, with an expected ASR of 0.5 in this typically monogamous genus. The distribution of 26 adult sex ratios across 12 species was normal (Shapiro-Wilkes, W = 0.98, p = 0.73), and deviation from 0.05 was tested with a 2 tailed Z-Test, using the standard deviation of adult sex ratios collected from the range of studies. Statistical analysis conducted using JMP Pro 11.2.0 (SAS Institute Inc.).

## Results

### Patterns of activity differ between density treatments

**Fig 1:**
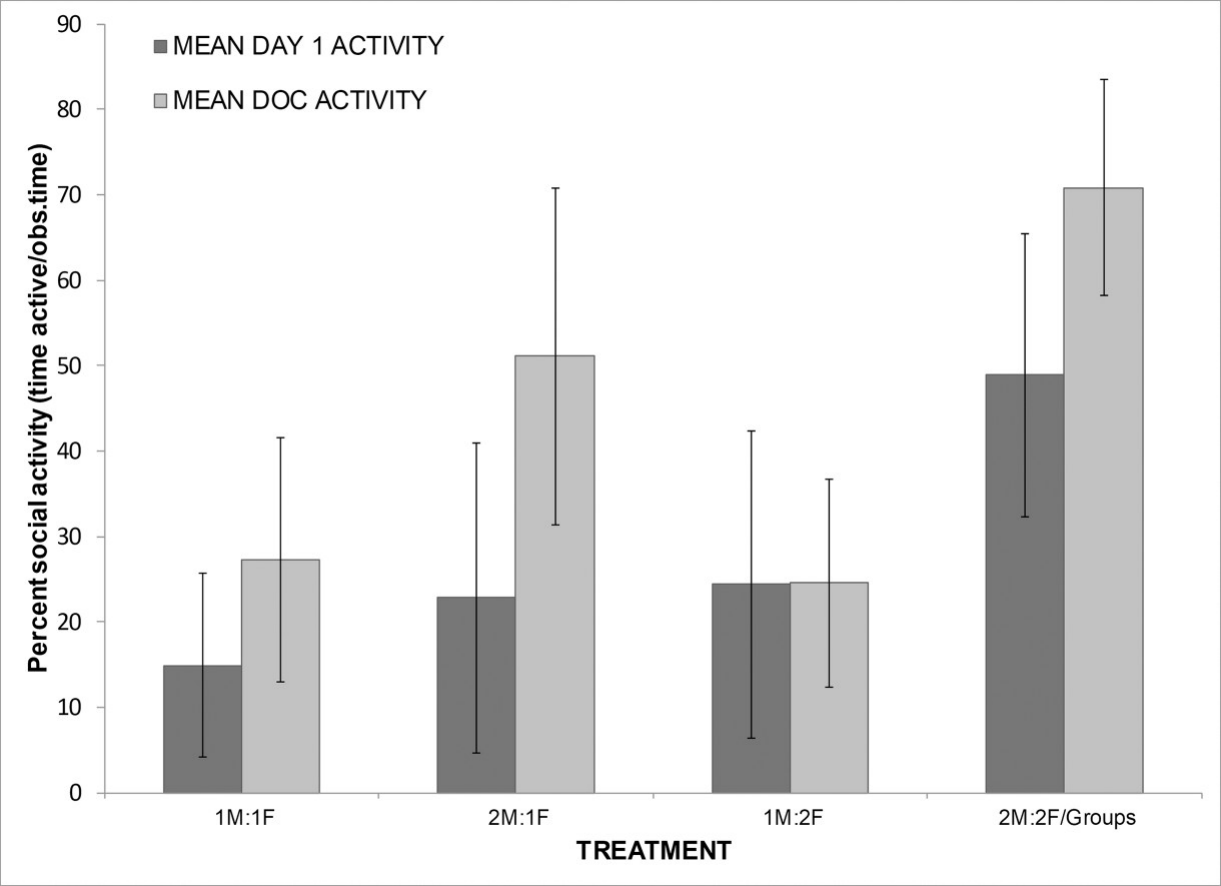
Activity level in tanks over time. Percent of time (±SD) *H*. *zosterae* spent engaged in social behaviors (courtship or competitive behaviors) on first day of courtship (Day 1) and day of copulation (DOC) calculated as time engaged in courtship/competition per total observation time.

**Fig 2:**
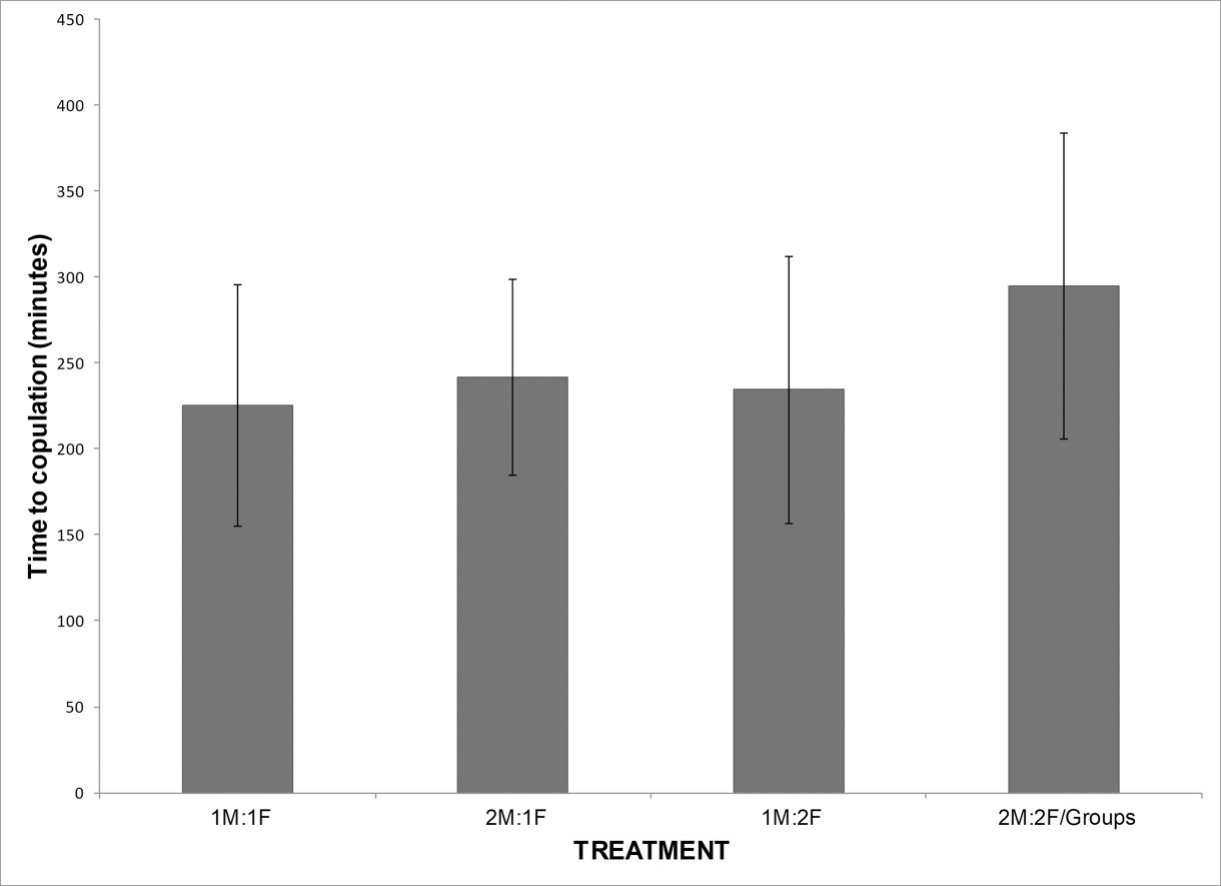
Differences in time to copulation between treatments. Time to copulation (±SD) in minutes on the day of copulation for *H*. *zosterae* pairs from treatments varying in density: 1M:1F (14.6 animals m^-2^), 1M:2F (21.9 animals m^-2^), 2M:1F (21.9 animals m^-2^, and high density (42.8 animals m^-2^).

Stocking density treatments varied significantly in activity level, both on the first day of courtship (Kruskal-Wallis, χ^2^ = 20.50, 3 df, p<0.0001; Fig 1) and the day of copulation (ANOVA, F_3,72_ = 12.71, p<0.0001). On the first day of courtship, increasing density from 14.6 to 42.8 animals per m^2^ was correlated with an increase in overall social activity of 228%. This pattern was also marked on the day of copulation, when the rate of social activity increased by 160% (Dunnett’s test with 1:1 treatment as control, both 2:1 and high density group p<0.05). In addition, not only was there a significantly higher rate of activity on the day of copulation at high densities, but the activity persisted for a longer period of time, increasing the time until copulation by 31% (ANOVA, F_3,77_ = 7.09, p = 0.0003, Dunnett’s test comparing treatments to 1:1 control, high density treatment p<0.05; Fig 2). Higher density tanks displayed a higher activity level and a longer time active on the day of copulation, but they also displayed a significantly larger number of fish who were on average engaged in courtship or competitive activities per active time, indicating broader social interactions than just the copulating pair (Kruskal-Wallis, Day 1: χ^2^ = 16.59, 3 df, p = 0.0009, DOC: χ^2^ = 15.01, 3 df, p = 0.002; Table 2). For example, high density tanks displayed an increase in time to copulation of 31%, compared to the 1:1 control tanks, and over the active time on that day, an average of 3 fish were engaged in courtship or competitive behaviors, compared to the lowest density treatments which had a shorter day of copulation and an average of only 1.5 fish active per social interaction.

**Table 2:** Mating behavior across density treatments.

TREATMENT	1:1	1:2	2:1	2:2/MULTI
**Sample size**	35	14	18	17
**Mean density (animals/m2)**	14.6	21.9	21.9	42.8
**Mean male density (males/m2)**	7.3	7.3	14.8	20.9
**DAY 1—Frequency of aggressive interactions (SD; # interactions/hour observation time/per male)**	0.54(0.22)	1.05(0.24)	0.77(0.24)	2.66(0.41)
**DAY 1 –Mean number of fish interacting**	1.58(0.19)	1.52(0.28)	1.65(0.26)	3.09(0.50)
**DAY 1—Mean # courtship partners—males**	1	1.28	1	2.12
**DAY 1—Mean # courtship partners—females**	1	1	1.067	1.8
**DOC—Frequency of aggressive interactions (SD; # interactions/hour observation time/per male)**	0.54(0.38)	1.56(0.41)	2.64(0.40)	2.46(0.70)
**DOC–Mean # of fish interacting**	1.53(0.15)	1.72(0.38)	1.95(0.38)	3.04(0.98)
**DOC—Mean # courtship partners—males**	1	1.71	1	2
**DOC—Mean # courtship partners—females**	1	1	1.33	1.7
**DOC—Percent of trials with transfer of eggs**	94.3	92.3	66.7	75.0
**DOC—Percent of trials with egg transfer errors**	5.7	7.1	66.7	76.4
**Percent of trials in which males dropped brood**	15	0	40	46.1
**Mean brood size (SD)**	16.9(13.3)	17.3(2.3)	14.3(8.0)	12.5(3.5)

**Mating behavior across density treatments**, including number of replicates in each treatment, mean animal and male density (SD; animal/male per m^2^), for both the first day of courtship (Day1) and the day of copulation (DOC)frequency of aggressive interactions (H,W,S), and mean number of courtship partners for males and females; percent of trials that had successful egg transfer, number of trials in which embryos rejected, and mean (SD) brood size for those treatments that resulted in successful offspring.

### Behavioral patterns differ between density treatments

Increasing density has general behavioral effects independent of sex, but because males are the more competitive sex for mating partners in seahorses [26,38], it is appropriate to investigate the frequency of aggressive behaviors relative to the density of males (Table 3). Both on the first day of courtship and on the day of copulation, the rate of aggressive behaviors (holding, wrestling, snapping) increased with increasing density of males (Day 1: Kruskal-Wallis test, χ^2^ = 10.81, 3 df, p = 0.013; DOC: χ^2^ = 15.94, 3 df, p = 0.001; Table 3). The display of specific competitive behaviors varied with treatment as well, with wrestling observed more commonly the more males there were in a treatment both the first day of courtship (Contingency Table Analysis: χ^2^ = 29.70, 1 df, p<0.0001; Table 3) and the day of copulation (CTA: χ^2^ = 44.50, 1 df, p<0.0001). Snapping showed the same trend, never observed in treatments with single males, but observed with increasing consistency in trials with more males on both the first day of courtship (CTA: χ^2^ = 30.50, 1 df, p<0.0001) and the day of copulation (CTA: χ^2^ = 50.70, 1 df, p<0.0001). Both wrestling and snapping were initiated only by males in treatments. Holding, a behavior observed in all treatments, was seen more frequently in trials with more males both early in courtship (CTA: χ^2^ = 18.21, 1 df, p = 0.0004) and later in courtship (CTA: χ^2^ = 53.70, 1 df, p<0.0001). Pumping is a behavior seen in 100% of trials on the day of copulation, but only seen infrequently on the early days of courtship in lower density tanks with fewer males, and seen more commonly in early courtship in trials that had higher numbers of males (CTA: χ^2^ = 10.04, 1 df, p = 0.018).

**Table 3:** Behaviors observed across density treatments.

TREATMENT	PERCENT OF TRIALS IN WHICH BEHAVIORS OBSERVED									
	FIRST DAY OF COURTSHIP					DAY OF COPULATION				
	Q	H	W	S	P	QPR	H	W	S	OTHER
1:1	100	53	4	0	27	100	20	0	0	0
1:2	100	71	21	0	14	100	76	54	23	38*
2:1	100	94	31	12	33	100	100	56	61	22+
2:2/MULTI	100	93	78	64	65	100	100	75	72	45+,38#

**Behaviors observed across density treatments** on both the first day of courtship and the day of copulation, with the percent of trials in which the behavior was observed indicated. Quivering (Q), Quivering, Pointing, and Rising (QPR) Holding (H), Wrestling (W), Snapping (S), and Pumping (P) are behaviors indicative of either early courtship, courtship ending in egg transfer, or competition.

* indicates trials in which both females pointe

+ indicates trials in which males rose together in water column

# indicates trials where males dropped clutch because interacting with a mating couple.

Patterns of mating behavior differed as well across treatments, with the least flexibility in mating partners seen in 1:1 treatments where there was the constraint of only one other individual to court. However, as animal density increased, on both the first day of courtship and the day of copulation, both males and females courted available members of the opposite sex, with increasing numbers of courting partners with increasing density of members of the opposite sex (Kruskal-Wallis tests: Males-Day 1 - χ^2^ = 31.20, 1 df, p<0.0001; Males-DOC - χ^2^ = 36.50, 1 df, p<0.0001; Females-Day 1 - χ^2^ = 30.70, 1 df, p<0.0001, Females-DOC - χ^2^ = 21.40, 1 df, p<0.0001; Table 2).

### Mating success differs between treatments

Between treatments, mating success (transfer of any eggs from female to male) was highest in low density treatments with fewest males and lowest in the higher density treatments with more males (CTA: χ^2^ = 13.61, 1 df, p = 0.004; Table 2), most likely because competing males can cause each other to miss aligning with the female during the copulatory transfer of eggs. The frequency of partial egg transfers (some eggs dropped in the process but at least some transferred from the female to the males’ pouch) also increases with increasing male density, with roughly 50% of trials in which at least 2 males are competing for females resulting in partial transfers (CTA: χ^2^ = 33.80, 1 df, p<0.0001). Finally, although not significant because of small sample sizes, the percent of trials in which males drop clutches before birth increases with increasing animal density, and brood sizes in larger groups are smaller (Table 2). These dropped clutches are frequently associated with brooding males engaging in competition with other males for females ready to transfer eggs, because pregnant males were rarely observed to pump and eject a clutch in trials with only one male (5 of 43 trials), but have been observed in 33% 2:1 trials (2 of 6 trials observed post-copulation) and 50% of higher density groups (7 of 14 trials observed post-copulation) engaging in competition, leading to pouch pumping and the ejection of embryos from the pouch in some of those males to allow for the male to receive a new and potentially larger brood of eggs (CTA: χ^2^ = 14.20, 2 df, p = 0.0008).

### Seahorse population biology meta-analysis

Mean wild population densities of seahorse studies range from 0.0014 ind m^-2^ in *H*. *mohnikei* to 0.241 ind m^-2^ in *H*. *guttulatus* (species $\bar{X}$ 0.064 (0.022) ind m^-2^, n = 14 species; S1 Table), with occasional local population maxima observed in the wild of up to 6 ind m^-2^ seen in natural habitats (*H*. *capensis* [42]; mean natural 0.081 (0.031) ind m^-2^, n = 39 studies) up to 13.1 ind m^-2^ seen in artificial habitats (*H*. *guttulatus* [43]; $\bar{X}$ artificial 0.227 (0.111) ind m^-2^, n = 9 studies). Across 30 studies surveyed, the adult sex ratio (ratio of males to total adults) was 0.457 (0.017), significantly biased towards females (Z-test, Z = -2.48, df = 29, p = 0.013 [2-tailed]).

Natural and artificial habitats across all studies surveyed differed significantly, with seahorses congregating at higher densities in human altered landscapes (S1 Table, Wilcoxon test: X^2^ = 4.71, 1 df, p = 0.03). Sampling methods also were associated with differences in reported densities, with focal studies and mark recapture studies ($\bar{X}$: 0.198 (0.090) ind m^-2^, n = 13) resulting in significantly higher densities than studies employing random or haphazard belt transect methods ($\bar{X}$: 0.03 (0.008) ind m^-2^, n = 26; Wilcoxon test: X^2^ = 10.36, 1 df, p = 0.001; S1 Table) even when matched for species represented in each group (focal: n = 11, belt: n = 18; Wilcoxon test: X^2^ = 4.28, 1 df, p = 0.039).

## Discussion

### Effects of stocking density on behavior

Overall, increasing the density of fish in tanks, and specifically males in tanks, significantly increased activity level, increased the mean number of fish engaged in courtship/competitive interactions per active time, led to higher levels of competition, a significantly higher rate of either fully failed or partial brood transfer from female to male due to male competition leading to eggs dropped on the tank bottom, and an increased rate of pregnancy failure due to pumping to eject the clutch in multi-male treatments. This increase in activity level on a per fish basis likely translates into higher metabolic demands for animals, similar to the increasing metabolic rates observed for male and female *H*. *zosterae* from the first day of courtship to the day of copulation [44]. Thus, observations of increased activity levels in courtship, copulation, and competition translate to increased metabolic costs for males and females.

Increased metabolic demands associated with higher activity may be one reason why a trend may exist for higher levels of pregnancy failure with increasing male densities; however, this is an area that needs focus for future research. Although metabolic rate increases for males and females on the day of copulation, for pregnant males, metabolic rate increases from 10–52% above pre-gravid levels during pregnancy [44]. To compensate for metabolic costs, males may be absorbing the nutrients from the broods in their pouches, similar to the process that has been observed in two species of pipefish (*Syngnathus typhle* [45]; *S*. *scovelli* [46]). In addition, in these higher density tanks, brooding males have been observed in at least 50% of trials engaging in competition with other males for females, and have been observed to eject embryos while “pumping”, which indicates his readiness to mate to the female, and thus leading to the observed higher pregnancy failure rates in these treatments. This pattern has not been described in other seahorse species to the authors’ knowledge, but biologically, this is a similar phenomenon to the partial brood reduction reported in pipefish [46,47], but here the entire brood is aborted. The ejection of a current brood in favor of another mating opportunity is an example of a behavior with potentially questionable adaptive value, either in the wild or captivity, especially if it occurs after substantial investment in the current brood or if the subsequent mate is smaller than the original mate and thus has smaller or fewer eggs to transfer. However, this is similar to the Bruce Effect, observed in both laboratory and wild mice and primates, in which females terminate pregnancies in response to the odors of unfamiliar males or in male-dominated, unstable groups, to theoretically prevent investing in offspring that will be killed if a new dominant male takes over the group [48–50].

### Recommendations for aquaculture/aquarium husbandry

Because of the high variability in tank shape/dimensions and stocking densities of animals -in public aquaria, a survey was conducted of studies describing seahorse aquaculture to help further understand the implications of high stocking densities (Table 1). Most studies of this type focus on juvenile growth and survival in culture, but a few have worked with determining optimal brood-stock conditions for juvenile production. Segregation by sex has been investigated as a tool to improve growth in *H*. *abdominalis* [31], but keeping animals separate or in 1:1 sex ratios had no effect on growth. This may be because animals were prevented from breeding by the tank design, but also could be that sex-segregated tanks also displayed courtship behaviors, and thus may have had equal activity levels relative to the stocking density (which was kept constant).

Although it is obviously not feasible to reduce aquarium environments to stocking densities equivalent to those observed in the field, we can take steps to reduce the impact of higher densities on the welfare of animals in human care. First, based on the present work and the research of others [31,34,51], it is recommended that holding tanks be maintained at a 1:1 sex ratio or a slightly female biased sex ratio. Fewer males relative to females will result in fewer competitive behaviors overall, in addition to reduced activity levels, translating into potentially lower food costs. Adequate anchor sites spread throughout tanks may also help as well, to reduce the need for animals to aggregate at few attachment sites, thus fostering more negative contact and potentially aggressive displays. Aquaculture design could also include “hiding spots” that allow fish to escape from intense social interactions, especially females who were often the targets of male aggression in *H*. *zosterae*.

In operations meant for breeding, pairs should be isolated from others (as seen [32,35]), either in separate tanks or by using mesh to separate pairs to reduce interactions with other fish. This will reduce the stress associated with aggressive behaviors as well as decrease the rate of dropped or partial clutch transfer because of competing males. However, if effective tank size is small relative to the number of fish contained, particular attention should be paid to water quality issues, because studies suggest that in pipefish, chemical cues can be used to identify males and females [52]. Although untested in seahorses, because of their close phylogenetic relationship and similar habitats, it is reasonable to consider that animals housed in large flow-through systems can detect each other, thus potentially ramping up competition even further.

### Comparison of *in situ* and *ex situ* behaviors in seahorses

Because of their small size and complex seagrass habitats, field observations of *H*. *zosterae* behavior are difficult at best. However, there are a growing number of studies of mating behavior in the field with larger species that can provide context to the range and intensity of competitive behaviors observed in dwarf seahorses in human care (Table 4). At the high *ex situ* densities in both the present study and with *H*. *fuscus* [38], competitive behaviors were seen at very high frequencies (in up to 87% of trials). However, with species such as *H*. *breviceps* [53], *H*. *guttulatus* [54] and *H*. *whitei* [9] found at higher wild densities, the rates of aggression in the wild are exceedingly low, observed in 1–8% of interactions. It is possible, however, that there are some species for which higher field densities occur, and thus social interactions and competition are more common; in *H*. *guttulatus*, local densities have been reported up to 10 animals m^-2^ [8], and extra-pair interactions are frequent [54]. In captivity, *H*. *guttulatus* have been observed to engage in higher levels of competitive behaviors associated with an increasing density of males [34]. Overall, whether it can be attributed to the lower density of field populations or other factors, the existence of high levels of competition in aquarium environments is a husbandry issue we must consider, using cues from what we know of the field biology of animals.

**Table 4:** Comtparison of field and *ex situ* behaviors observed.

Species	Study Type	Max Density	ASR	Behaviors	Ref
***H*. *abdominalis***	field	0.2	0.29	F-F interference; Males courted multiple mates; Males smaller than females	[14]
***H*. *abdominalis***	field	0.01	0.31	Most seen alone (60–75%), but groups observed up to 10	[55]
***H*. *breviceps***	field	0.7	0.47	Snapping, aggression observed twice in 25 days with 38 animals– **8% aggression rate**	[53]
***H*. *capensis***	field	0.46	0.48	No courtship or aggressive behaviors observed; nearby animals all M-F or F-F groups, in 91 animals	[18]
***H*. *comes***	field	4 animals/group	0.49	No aggression described	[56]
***H*. *fuscus***	*ex situ*	6.6	0.66/0.33	Snapping observed in 13/15 trials (**87%**); Wrestling in 4/15 trials (**27%**)	[38]
***H*. *guttulatus***	field	1.52	0.45	Of approximately 293 recorded greetings/courtship bouts, 11 pairs interrupted by a male, 10 of those resulting in snapping, wrestling, and chasing– **3.7% aggression rate**	[54,57]
***H*. *guttulatus***	*ex situ*	1.52	0.5/.75/0.25	Competition occurred in 29% of courtship interactions	[34]
***H*. *guttulatus***	*ex situ*	58	0.39/0.5/0.63	Genetic analysis shows mate switching both between and within seasons; mate switching more likely in male-biased (50%) and unbiased (37.5%) treatments	[13]
***H*. *reidi***	field	0.045	0.53	Only M-F reproductive behavior observed; 78% of grouping behavior 1M:1F, 20% 3 seahorses, groups above 4 rare	[58]
***H*. *subelongatus***	field	0.016	0.42	Mate switching between broods observed in 43% of pairs	[11,59]
***H*. *whitei***	field	0.215	0.48	In 33 pairs, 95 greetings observed and only one instance of MM-aggression (snapping) was observed; **1% aggression rate**	[9,60,61]
***H*. *zosterae***	field	1.54 /3.33	0.30 /0.54	No behavior described	[21]
***H*. *zosterae***	*ex situ*	21/29	0.66/0.33	Larger /M-biased groups = 100% Holding, 75% Wrestling, 72% Snapping, 45% MM rises, 38% M dropped clutch	present study

In addition to the increased aggressive behaviors of males competing in higher densities in captivity, we also see changes in their reproductive behaviors. Reports of decreased reproductive success from high densities in husbandry may be a concern but they pose an even greater threat when it comes to the natural populations. As a result of seahorses losing their habitats, populations’ sizes are expected to decrease [62]. However, populations would also be predicted to become locally denser as a result of habitat loss or reliance on artificial habitats concentrating seahorses displaced from surrounding areas, which has been documented in Portugal, Australia and South Africa [43,63,64]. Increased densities have been shown to lead to mate switching in the present study, with increased rates of rejection of embryos from a male’s pouch during pumping behaviors resulting in failed pregnancies. This has the potential to decrease the number of pregnancies compared to monogamous pairings, and allows male to remate with new females. Previous research has shown as densities increase the correlation between mating success and postmating sexual selection mechanisms, such as fertilization success or embryo survivorship, weaken [65]. This rejection of developing embryos results in females that were successful mating to have zero reproductive success potentially altering the strength of sexual selection in high density populations. Brood removal during a pregnancy is a waste of resources, time, and energy for both sexes because the developing embryos cannot survive outside the male’s pouch and ultimately can lead to a lower number of offspring throughout the breeding season.

Male-biased sex ratios can also impact the reproductive output of natural populations as a result of male aggression leading to unsuccessful egg transfers and partial pregnancies. Although the mean sex ratio across all of the studies surveyed was female biased, one fourth of the studies were male biased (although only one significantly so, S1 Table). If availability of suitable habitat decreases then high local densities can lead to increased interactions among males, even in female biased populations. As seahorses congregate on limited vegetation or even preferred artificial habitats there is a potential for more male aggression leading lower mating successes. Even extremely female biased populations have shown evidence of altered courtship behavior, with *H*. *abdominalis* observed in highly female dominated sex ratios demonstrating female interference and males courting multiple females. Although monogamy had been confirmed within broods for a number of species [10–15], mate switching between broods has the potential to lower overall reproductive rates, impacting females more than males due to the structure of female ovaries and limitations in egg production [24,25,66].

### Meta-analysis of seahorse populations worldwide

Field research investigating seahorse populations in the wild indicates that these animals are found at low mean densities which vary over time and space naturally ($\bar{X}$ = 0.064 (0.022) seahorses m^-2^ calculated across 14 seahorse species; S1 Table). However, due to methodological differences between these studies, this mean density has little meaning for understanding the context of animals in captivity, especially comparing visual census to direct sampling methods (seine, pushnet, etc; [67]). To illustrate, field studies from *H*. *zosterae* have been conducted using three different techniques, short pushnet transects [21], long pushnet transects [19, 21], and a combination of focal study grids and mark-recapture [21]. All three techniques have yielded different field densities, from 0.067 using focal study grids to 0.189 using short pushnet transects (first numbers provided, S1 Table; [8]). In addition, these mean densities include, as do the mean densities of many studies in S1 Table, transects in which no seahorses were observed. These “zero s” are essential to include for management of seahorse species because they help us describe the landscape-level low density of these animals [17,68–69], but their inclusion hinders our ability to understand their biology on a local scale. Excluding zeros from the analysis more than doubles the measured density for focal study grids and short pushnet samples, but does little to improve the resolution of longer transect samples (second numbers, S1 Table). Of critical importance for understanding the wild biology is the local density of animals, provided through both non-zero samples and reported maximum or group density measures. For *H*. *zosterae*, maximum densities reported are between 1.5–3.3 animals/m^2^, below the range of 14–42 animals/m^2^ used in this captive study. In future field studies, it is strongly recommended that locally high densities be reported along with mean densities, to better understand the context of wild animals and those in captivity.

## Conclusions

To better understand the impact of population densities on mating behaviors and determine their effects on the reproductive output of seahorses, more natural populations will need to be surveyed. There is also need for studies to determine the causes behind the variation in densities across species worldwide and whether they are a result of variation in survey methods, interspecies variations, methodology for calculating seahorse density, the particular type of habitats that are being surveyed and when for each study, or from differences in the impacts of anthropogenic factors [70]. From a conservation perspective, it is important that when using seahorse densities for determining whether or not all species of seahorses should be included in the IUCN Red List, the sampling techniques used for each study must be considered. Ultimately, in this study we have demonstrated the importance of understanding how the behavioral changes across densities and sex ratios might be important for the success of these species and outline some of the challenges associated with placing *ex situ* studies in the context of natural population densities of seahorses.

## Supporting information

S1 Table Survey of field studies conducted on seahorse species worldwide, investigating seahorse sample size (n), mean animal density (animals m^-2^; provided directly from reference or calculated if not provided; first number in column used in overall calculations), maximum animal density (animals m^-2^), adult sex ratio (Males:Total Animals; * indicates significant deviation from unity), and sampling method. Studies were excluded if there was no way to convert either group numbers or density measurement variables to animals m^-2^. Two numeric values provided either indicate that the study had two different methods presented (sampling method), investigated natural and artificial habitats, or data are provided for distant locations. NR = Not Reported, ^+^ indicates artificial habitat values.
(DOCX)

S1 Fig Male on left image snapping at male with fully inflated pouch on far right of image, over the top of the female they are both courting in the middle image. (JPG)

S2 Fig Male on right image holding the tail of the female in the middle of the image, with the other competing male on the far left of the image. (JPG)

S3 Fig Two males (left and middle foreground image) holding onto 2 females (right and middle background image), who are holding on to each other and all struggling to separate.
(JPG)

S4 Fig Just prior to image, male on left image and female (right image) were courting, and the male in the middle image intruded into their courtship.
(JPG)

S5 Fig Reciprocal quivering displayed, with male in the foreground and female in the background. Notice the erect body posture and the darkened dorsal line but bright abdomen and latera surface of the male and female.
(JPG)

S6 Fig Newly gravid male, with eggs transferred within the previous minute from the female to the male. (JPG)

S1 Video Four males and 4 females are displayed in a 38 l tank, with 1 male carrying embryos from a previous egg transfer. Time counter in upper left screen corresponds to times detailed in the video annotations that occur in the side panels as the film moves forward.
(ZIP)
