# Supplementary material for: When more is not merrier: Using wild population dynamics to understand the effect of density on ex situ seahorse mating behaviors
Source: PLoS One. 2019 Jul 2;14(7):e0218069. doi: 10.1371/journal.pone.0218069 (PMC6605648; doi:10.1371/journal.pone.0218069)
Supplement: S1 Table — Studies were excluded if there was no way to convert either group numbers or density measurement variables to animals m-2. Two numeric values provided either indicate that the study had two different methods presented (sampling method), investigated natural and artificial habitats, or data are provided for distant locations. NR = Not Reported, + indicates artificial habitat values. (DOCX) [file pone.0218069.s001.docx]

**S1 Table: Field studies conducted on seahorse species worldwide**

| **Species** | **Mean Density (#** m^-2^**)** | **Max Density (#** m^-2^**)** | **ASR** | **Sampling method** | **Ref** |  |
| --- | --- | --- | --- | --- | --- | --- |
| ***H. abdominalis (n=902)*** | 0.010 | NR | 0.31* | Stratified random belt transects | [[1]](https://paperpile.com/c/HBGxF8/aXrB) |  |
|  |  |  |  |  |  |  |
| ***H. abdominalis (n=112)*** | 0.067 | 0.200 | 0.29* | Opportunistic observation | [[2]](https://paperpile.com/c/HBGxF8/OJMfS) |  |
| ***H. abdominalis (n=51)*** | 0.035^+^ | 0.065^+^ | 0.61 | Sampling of Beach Nets | [[3]](https://paperpile.com/c/HBGxF8/jFJq) |  |
| ***H. breviceps (n=38)*** | 0.210 | 5 animals/group | 0.47 | Focal study grids | [[4]](https://paperpile.com/c/HBGxF8/YS4sN) |  |
| ***H. capensis (n=44,91)*** | 0.0089/0.220 | 0.25/0.460 | 0.71*/0.48 | Transect/focal study grids | [[5]](https://paperpile.com/c/HBGxF8/FLn6) |  |
| ***H. capensis (n=452)*** | 0.015 | 6.0 | NR | Random belt transects | [[6]](https://paperpile.com/c/HBGxF8/1xIO) |  |
| ***H. capensis (n=278)*** | 0.008 | NR | NR | Random belt transects | [[7]](https://paperpile.com/c/HBGxF8/xZiw) |  |
| ***H. capensis (n=484)*** | 0.020/0.156^+^ | 0.110/0.640 | 0.43 | Random belt transects; Natural/Artificial | [[8]](https://paperpile.com/c/HBGxF8/jnkNS) |  |
| ***H. capensis (n=669)*** | 0.035/0.260^+^ | NR | 0.30* | Random belt transects; Natural/Artificial | [[9]](https://paperpile.com/c/HBGxF8/kJRGu) |  |
| ***H. comes (n=32)*** | 0.0054 | 0.019 | 0.49 | Focal study grids | [[10]](https://paperpile.com/c/HBGxF8/mNjA) |  |
| ***H. comes (n=328)*** | 0.0014/0.0008^+^ | 0.0022 | 0.47 | Random belt transects; Natural/Artificial | [[11]](https://paperpile.com/c/HBGxF8/iXPJ) |  |
| ***H. coronatus (n=555)*** | 0.0029 | 0.0065 | 0.38* | Seine net | [[12]](https://paperpile.com/c/HBGxF8/vz5G) |  |
| ***H. erectus (n=NR)*** | 0.0008 | NR | NR | Random epibenthic sleds | [13] |  |
| ***H. erectus (n=5)*** | 0.0004 | NR | NR | Long pushnet transects | [14] |  |
| ***H. erectus (n=NR)*** | 0.0098 | NR | NR | Rollerframe trawls | [15] |  |
| ***H. guttulatus (n=384)*** | 0.073 | 0.51 | NR | Random belt transects | [[16]](https://paperpile.com/c/HBGxF8/1jpt) |  |
| ***H. guttulatus (n=700)*** | 0.62 | 1.5 | 0.45 | Focal study grids | [[17,18]](https://paperpile.com/c/HBGxF8/zlQIJ+58dm) |  |
| ***H. guttulatus (n=58)*** | 0.004 | 0.035 | 0.42 | Random belt transects | [[19]](https://paperpile.com/c/HBGxF8/IonM) |  |
| ***H. guttulatus (n=225)*** | 0.018/0.019^+^ | 0.035 | 0.46 | Random belt transects; Natural and Artificial | [[20]](https://paperpile.com/c/HBGxF8/iU8P) |  |
| ***H. guttulatus (n=317)*** | 0.041/0.165^+^ | 0.080 | 0.52 | Random belt transects; Natural and Artificial | [[21]](https://paperpile.com/c/HBGxF8/PHyl) |  |
| ***H. guttulatus (n=1675)*** | 0.107 | 0.508 | NR | Random belt transects | [[22]](https://paperpile.com/c/HBGxF8/JdZ4T) |  |
| ***H. hippocampus (n=41)*** | 0.007 | 0.072 | NR | Random belt transects | [[16]](https://paperpile.com/c/HBGxF8/1jpt) |  |
| ***H. hippocampus (n=18)*** | 0.001 | 0.019 | 0.62 | Random belt transects | [[19]](https://paperpile.com/c/HBGxF8/IonM) |  |
| ***H. hippocampus (n=17)*** | 0.0005 | 0.003 | NR | Random belt transects | [[20]](https://paperpile.com/c/HBGxF8/iU8P) |  |
| ***H. mohnikei (n=252)*** | 0.0014 | 0.0082 | 0.38 | Seine net | [[12]](https://paperpile.com/c/HBGxF8/vz5G) |  |
| ***H. reidi (n=911)*** | 0.026 | 0.045 | 0.53 | Random belt transects | [[23]](https://paperpile.com/c/HBGxF8/Ax2B) |  |
| ***H. reidi (n=20)*** | 0.018 | 0.04 | 0.25 | Focal study grids | [[24]](https://paperpile.com/c/HBGxF8/e1tP) |  |
| ***H. reidi (n=38)*** | 0.01187 | 0.044 | 0.50 | Belt transects | [[25]](https://paperpile.com/c/HBGxF8/x731) |  |
| ***H. reidi (n=111)*** | 0.010 | 0.017 | 0.50 | Mark-recapture/Focal study grids | [[26]](https://paperpile.com/c/HBGxF8/P8Cb) |  |
| ***H. reidi (n=57)*** | 0.031 | 0.1 | 0.5 | Focal study grids | [[27]](https://paperpile.com/c/HBGxF8/R1ta) |  |
| ***H. subelongatus (n=216)*** | 0.011 | 0.0158 | 0.42 | Focal study grids | [[28]](https://paperpile.com/c/HBGxF8/kJ1gH+ubi6) |  |
| ***H. whitei (n=98)*** | 0.152 | 0.215 | 0.48 | Focal study grids | [[[29,30,31]](https://paperpile.com/c/HBGxF8/JqdTH+IrWb+oca7) |  |
| ***H. whitei (n=886,314)*** | 0.072/1.05^+^ | NR | NR | Mark-Recapture; Natural and Artificial | [[32]](https://paperpile.com/c/HBGxF8/XfsX) |  |
| ***H. whitei (n=498)*** | 0.343^+^ | 1.0 | 0.56* | Sampling of Beach Nets; Artificial | [[33]](https://paperpile.com/c/HBGxF8/jFJq) |  |
| ***H. whitei (n=1527)*** | 0.029 | NR | NR | Mark-Recapture; Natural and Artificial | [[34]](https://paperpile.com/c/HBGxF8/3nbI) |  |
| ***H. whitei (n=317)*** | 0.041 | 0.187 | 0.45 | Belt transects | [[35]](https://paperpile.com/c/HBGxF8/42hq) |  |
| ***H. zosterae (n=70,58)*** | 0.189; 0.189(0.32)/0.472(0.31) (FK); 0.126 (0.40) / 0.663(0.68) (TB) | 1.54 (FL Keys); 3.33 (Tampa ) | 0.30* (FL Keys); 0.54 (Tampa) | Short pushnet transects | [[36]](https://paperpile.com/c/HBGxF8/qbvV5) |  |
| ***H. zosterae (n=922)*** | 0.084; 0.084(0.11)/0.091(0.11) | 0.535 | 0.41* | Long pushnet transects | [[14]](https://paperpile.com/c/HBGxF8/Sdc3m) |  |
| ***H. zosterae (n=1037)*** | 0.139(0.02) | 1.25 | 0.38* | Long pushnet transects | [[37]](https://paperpile.com/c/HBGxF8/qbvV5) |  |
| ***H. zosterae (n=64)*** | 0.067; 0.067(0.04)/0.095(0.04) | 0.17 | 0.51 | Focal study grids; pushnet/Mark Recapture | [[36]](https://paperpile.com/c/HBGxF8/qbvV5) |  |

**Field studies conducted on seahorse species worldwide**, investigating seahorse sample size (n), mean animal density (animals m^-2^; provided directly from reference or calculated if not provided; first number in column used in overall calculations), maximum animal density (animals m^-2^), adult sex ratio (Males:Total Animals; * indicates significant deviation from unity), and sampling method. Studies were excluded if there was no way to convert either group numbers or density measurement variables to animals m^-2^. Two numeric values provided either indicate that the study had two different methods presented (sampling method), investigated natural and artificial habitats, or data are provided for distant locations. NR = Not Reported, ^+^ indicates artificial habitat values.

**References:**

1. [Martin-Smith KM, Vincent ACJ. Seahorse declines in the Derwent estuary, Tasmania in the absence of fishing pressure. Biol Conserv. 2005;123: 533–545. doi:](http://paperpile.com/b/P1bR23/pbKY3)[10.1016/j.biocon.2005.01.003](http://dx.doi.org/10.1016/j.biocon.2005.01.003)

2. [Wilson AB, Martin‐Smith KM. Genetic monogamy despite social promiscuity in the pot‐bellied seahorse (Hippocampus abdominalis). Mol Ecol. 2007; 16: 2345-2352](http://paperpile.com/b/P1bR23/C8SSf). <https://onlinelibrary.wiley.com/doi/abs/10.1111/j.1365-294X.2007.03243.x>

3. [Vincent ACJ, Foster SJ, Koldewey HJ. Conservation and management of seahorses and other Syngnathidae. J Fish Biol. 2011;78: 1681–1724. doi:](http://paperpile.com/b/P1bR23/QWlcm)[10.1111/j.1095-8649.2011.03003.x](http://dx.doi.org/10.1111/j.1095-8649.2011.03003.x)

4. [Moreau M-A, Vincent ACJ. Social structure and space use in a wild population of the Australian short-headed seahorse Hippocampus breviceps Peters, 1869. Mar Freshwater Res. 2004;55: 231–239. doi:](http://paperpile.com/b/P1bR23/HqEP8)[10.1071/MF03159](http://dx.doi.org/10.1071/MF03159)

5. [Bell EM, Lockyear JF, McPherson JM, Dale Marsden A, Vincent ACJ. First field studies of an endangered South African seahorse, Hippocampus capensis. Environ Biol Fishes. 2003;67: 35–46. doi:](http://paperpile.com/b/P1bR23/7NeNl)[10.1023/A:1024440717162](http://dx.doi.org/10.1023/A:1024440717162)

6. [Lockyear JF, Hecht T, Kaiser H, Teske PR. The distribution and abundance of the endangered Knysna seahorse Hippocampus capensis (Pisces: Syngnathidae) in South African estuaries. Afr J Aquat Sci. 2006;31: 275–283. doi:](http://paperpile.com/b/P1bR23/f01Ce)[10.2989/16085910609503897](http://dx.doi.org/10.2989/16085910609503897)

7. [Teske PR, Lockyear JF, Hecht T, Kaiser H. Does the endangered Knysna seahorse, Hippocampus capensis, have a preference for aquatic vegetation type, cover or height? Afr Zool. Zoological Society of Southern Africa; 2007;42: 23–30. doi:](http://paperpile.com/b/P1bR23/3eRAL)[10.3377/1562-7020(2007)42[23:DTEKSH]2.0.CO;2](http://dx.doi.org/10.3377/1562-7020(2007)42%5b23:DTEKSH%5d2.0.CO;2)

8. [Claassens L. An artificial water body provides habitat for an endangered estuarine seahorse species. Estuar Coast Shelf Sci. 2016;180: 1–10. doi:](http://paperpile.com/b/P1bR23/GW7rH)[10.1016/j.ecss.2016.06.011](http://dx.doi.org/10.1016/j.ecss.2016.06.011)

9. [Claassens L, Hodgson AN. Monthly population density and structure patterns of an endangered seahorse Hippocampus capensis: a comparison between natural and artificial habitats. J Fish Biol. 2018;101: 723-733. doi:](http://paperpile.com/b/P1bR23/IVDjT)[10.1111/jfb.13639](http://dx.doi.org/10.1111/jfb.13639)

10. [Perante NC, Pajaro MG, Meeuwig JJ, Vincent ACJ. Biology of a seahorse species, Hippocampus comes in the central Philippines. J Fish Biol. 2002;60: 821–837. doi:](http://paperpile.com/b/P1bR23/FRTVz)[10.1111/j.1095-8649.2002.tb02412.x](http://dx.doi.org/10.1111/j.1095-8649.2002.tb02412.x)

11. [Morgan SK, Vincent ACJ. The ontogeny of habitat associations in the tropical tiger tail seahorse Hippocampus comes Cantor, 1850. J Fish Biol. 2007;71: 701–724. doi:](http://paperpile.com/b/P1bR23/bxtwK)[10.1111/j.1095-8649.2007.01535.x](http://dx.doi.org/10.1111/j.1095-8649.2007.01535.x)

12. [Choi Y-U, Rho S, Park H-S, Kang D-H. Population characteristics of two seahorses, Hippocampus coronatus and Hippocampus mohnikei, around seagrass beds in the southern coastal waters of Korea. Ichthyol Res. 2012;59: 235–241. doi:](http://paperpile.com/b/P1bR23/e6ALh)[10.1007/s10228-012-0285-z](http://dx.doi.org/10.1007/s10228-012-0285-z)

13. [Powell AB, LaCroix MW, Cheshire RT (2002) An evaluation of northern Florida Bay as a nursery area for red drum, Sciaenops ocellatus, and other juvenile and small resident fishes. NOAA Technical Memorandum NMFS-SEFSC-485:1–29](http://paperpile.com/b/JStdxj/7vKUr). http://aquaticcommons.org/2138/

14. [Masonjones HD, Rose E, Mc Rae LB, Dixson DL. An examination of the population dynamics of syngnathid fishes within Tampa Bay, Florida, USA. Curr Zool. 2010;56: 118−133.](http://paperpile.com/b/P1bR23/l1eH3)

15. Stallings CD, Brower JP, Heinlein Loch JM, Mickle A. Catch comparison between otter and rollerframe trawls: Implications for sampling in seagrass beds. Fish Res. Elsevier; 2014;155: 177–184. doi:10.1016/j.fishres.2014.03.002

16. [Curtis J, Vincent A. Distribution of sympatric seahorse species along a gradient of habitat complexity in a seagrass-dominated community. Mar Ecol Prog Ser. 2005;291: 81–91. doi:](http://paperpile.com/b/P1bR23/JLYtk)[10.3354/meps291081](http://dx.doi.org/10.3354/meps291081)

17. [Naud M-J, Curtis JMR, Woodall LC, Gaspar MB. Mate choice, operational sex ratio, and social promiscuity in a wild population of the long-snouted seahorse Hippocampus guttulatus. Behav Ecol. 2009;20: 160–164. doi:](http://paperpile.com/b/P1bR23/TuQMA)[10.1093/beheco/arn128](http://dx.doi.org/10.1093/beheco/arn128)

18. [Curtis JMR, Vincent ACJ. Life history of an unusual marine fish: survival, growth and movement patterns of Hippocampus guttulatus Cuvier 1829. J Fish Biol. 2006;68: 707–733. doi:](http://paperpile.com/b/P1bR23/PML97)[10.1111/j.1095-8649.2006.00952.x](http://dx.doi.org/10.1111/j.1095-8649.2006.00952.x)

19. [Caldwell IR, Vincent ACJ. Revisiting two sympatric European seahorse species: apparent decline in the absence of exploitation. Aquat Conserv. 2012;22: 427–435. doi:](http://paperpile.com/b/P1bR23/vgbdj)[10.1002/aqc.2238](http://dx.doi.org/10.1002/aqc.2238)

20. [Gristina M, Cardone F, Carlucci R, Castellano L. Abundance, distribution and habitat preference of Hippocampus guttulatus and Hippocampus hippocampus in a semi‐enclosed central Mediterranean marine area. Mar Rundsch. 2015;36: 57–66.](http://paperpile.com/b/P1bR23/Hnyp7) <http://onlinelibrary.wiley.com/doi/10.1111/maec.12116/full>

21. [Gristina M, Cardone F, Desiderato A, Mucciolo S, Lazic T, Corriero G. Habitat use in juvenile and adult life stages of the sedentary fish Hippocampus guttulatus. Hydrobiologia. 2017;784: 9–19. doi:](http://paperpile.com/b/P1bR23/GTHC9)[10.1007/s10750-016-2818-3](http://dx.doi.org/10.1007/s10750-016-2818-3)

22. [Correia M, Koldewey HJ, Andrade JP, Esteves E, Palma J. Identifying key environmental variables of two seahorse species (Hippocampus guttulatus and Hippocampus hippocampus) in the Ria Formosa lagoon, South Portugal. Environ Biol Fishes. 2018;101: 1357–1367. doi:](http://paperpile.com/b/P1bR23/xQXuY)[10.1007/s10641-018-0782-7](http://dx.doi.org/10.1007/s10641-018-0782-7)

23. [Rosa IL, Oliveira TPR, Castro ALC, Moraes LE de S, Xavier JHA, Nottingham MC, et al. Population characteristics, space use and habitat associations of the seahorse Hippocampus reidi (Teleostei: Syngnathidae). Neotrop Ichthyol. Sociedade Brasileira de Ictiologia; 2007;5: 405–414. doi:](http://paperpile.com/b/P1bR23/UbiFr)[10.1590/S1679-62252007000300020](http://dx.doi.org/10.1590/S1679-62252007000300020)

24. [Freret-Meurer NV, Andreata JV. Field studies of a Brazilian seahorse population, Hippocampus reidi Ginsburg, 1933. Braz Arch Biol Technol. Tecpar; 2008;51: 543–551. doi:](http://paperpile.com/b/P1bR23/kUZny)[10.1590/S1516-89132008000400012](http://dx.doi.org/10.1590/S1516-89132008000400012)

25. [de Oliveira VM, Freret-Meurer NV. Vertical distribution of the seahorse Hippocampus reidi Ginsburg, 1933 in the Arraial do Cabo region, Rio de Janeiro, Brazil. Biotemas. Universidade Federal de Santa Catarina; 2012;25: 59–66. doi:](http://paperpile.com/b/P1bR23/KYIBt)[10.5007/2175-7925.2012v25n2p59](http://dx.doi.org/10.5007/2175-7925.2012v25n2p59)

26. [Siqueira AC, Quimbayo JP, Cantor M, Silveira RB, Daura-Jorge FG. Estimating population parameters of longsnout seahorses, Hippocampus reidi (Teleostei: Syngnathidae) through mark-recapture. Neotrop Ichthyol. 2017;1-15. doi:](http://paperpile.com/b/P1bR23/kYSWL)[10.1590/1982-0224-20170067](http://dx.doi.org/10.1590/1982-0224-20170067)

27. Freret-Meurer N, Fernández T, Okada N, Vaccani A. Population dynamics of the endangered seahorse Hippocampus reidi Ginsburg, 1933 in a tropical rocky reef habitat. Population. 2018;41: 345–356. <http://abc.museucienciesjournals.cat/files/ABC_41-2_pp_345-356.pdf>

28. [Kvarnemo C, Moore GI, Jones AG, Nelson WS, Avise JC. Monogamous pair bonds and mate switching in the Western Australian seahorse Hippocampus subelongatus. J Evol Biol. 2000;13: 882–888. doi:](http://paperpile.com/b/P1bR23/bINC4)[10.1046/j.1420-9101.2000.00228.x](http://dx.doi.org/10.1046/j.1420-9101.2000.00228.x)

29. [Vincent ACJ, Sadler LM. Faithful pair bonds in wild seahorses, Hippocampus whitei. Anim Behav. 1995;50: 1557–1569. doi:](http://paperpile.com/b/P1bR23/QC8oQ)[10.1016/0003-3472(95)80011-5](http://dx.doi.org/10.1016/0003-3472(95)80011-5)

30. [Vincent ACJ, Dale Marsden A, Evans KL, Sadler LM. Temporal and spatial opportunities for polygamy in a monogamous seahorse, Hippocampus whitei. Behaviour. 2004;141: 141–156. doi:](http://paperpile.com/b/P1bR23/PTPr6)[10.1163/156853904322890780](http://dx.doi.org/10.1163/156853904322890780)

31. [Vincent ACJ, Evans KL, Marsden AD. Home range behaviour of the monogamous Australian seahorse, Hippocampus. Environ Biol Fishes. 2005;72: 1–12. doi:](http://paperpile.com/b/P1bR23/fWS2n)[10.1007/s10641-004-4192-7](http://dx.doi.org/10.1007/s10641-004-4192-7)

32. [Harasti D, Martin-Smith K, Gladstone W. Population dynamics and life history of a geographically restricted seahorse, Hippocampus whitei. J Fish Biol. 2012;81: 1297–1314. doi:](http://paperpile.com/b/P1bR23/D4DiU)[10.1111/j.1095-8649.2012.03406.x](http://dx.doi.org/10.1111/j.1095-8649.2012.03406.x)

33. [Clynick BG. Harbour swimming nets: a novel habitat for seahorses. Aquat Conserv. 2008;18: 483–492. doi:](http://paperpile.com/b/P1bR23/ardyz)[10.1002/aqc.856](http://dx.doi.org/10.1002/aqc.856)

34. [Harasti D. Declining seahorse populations linked to loss of essential marine habitats. Mar Ecol Prog Ser. 2016;546: 173–181. doi:](http://paperpile.com/b/P1bR23/iGbeu)[10.3354/meps11619](http://dx.doi.org/10.3354/meps11619)

35. [Manning C. How is a family of sedentary marine fishes shaped by its habitats, prey, and predators? Vincent A, editor. Masters, University of British Columbia. 2017.](http://paperpile.com/b/P1bR23/rFCnD)

36. [Masonjones H, Hayashida-Boyles A, Pollom R. Hippocampus zosterae. e.T10089A46910143. In: The IUCN Red List of Threatened Species [Internet]. 2017 [cited Jun 2018].](http://paperpile.com/b/P1bR23/ey5vz) http://dx.doi.org/10.2305/IUCN.UK.2017-3.RLTS.T10089A46910143.en.

37. Rose E, Simmonds M, Hayashida‐Boyles AL, Masonjones HD. Seasonal and spatial variation in the reproductive biology of the dwarf seahorse Hippocampus zosterae. J Fish Biol. 2019; 1– 10. https://doi.org/10.1111/jfb.13975
