# Supplementary figures and images for: When more is not merrier: Using wild population dynamics to understand the effect of density on ex situ seahorse mating behaviors
Source: PLoS One. 2019 Jul 2;14(7):e0218069. doi: 10.1371/journal.pone.0218069 (PMC6605648; doi:10.1371/journal.pone.0218069)

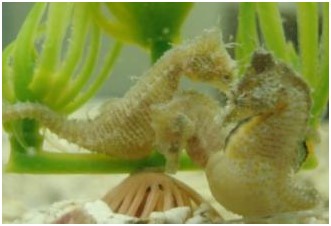

Supplement: S1 Fig — (JPG) [file pone.0218069.s002.jpg]

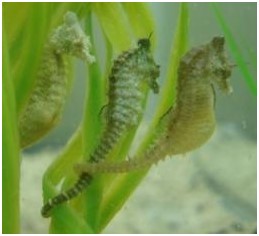

Supplement: S2 Fig — (JPG) [file pone.0218069.s003.jpg]

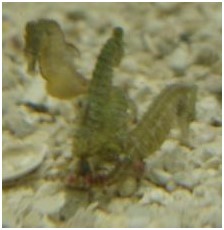

Supplement: S3 Fig — Two males (left and middle foreground image) holding onto 2 females (right and middle background image), who are holding on to each other and all struggling to separate. (JPG) [file pone.0218069.s004.jpg]

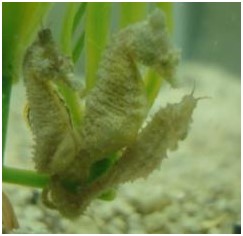

Supplement: S4 Fig — Just prior to image, male on left image and female (right image) were courting, and the male in the middle image intruded into their courtship. (JPG) [file pone.0218069.s005.jpg]

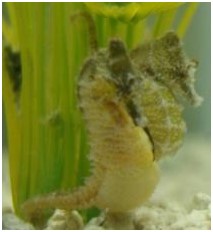

Supplement: S5 Fig — Notice the erect body posture and the darkened dorsal line but bright abdomen and latera surface of the male and female. (JPG) [file pone.0218069.s006.jpg]

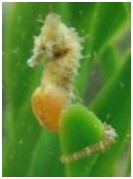

Supplement: S6 Fig — (JPG) [file pone.0218069.s007.jpg]
